# Research note: a resting-state, cerebello-amygdaloid intrinsically connected network

**DOI:** 10.1186/s40673-018-0083-0

**Published:** 2018-02-14

**Authors:** Christophe Habas

**Affiliations:** Service de NeuroImagerie, CHNO des, 15-20 Paris, France

**Keywords:** Cerebellum, Dendate nucleus, Amygdala, Insula, Parietal opercule, Resting-state, fMRI

## Abstract

**Background:**

Previous ROI-based functional connectivity studies found functional coherence between cerebellum and cerebral amygdale, at rest. Moreover, some neurospychiatric symptoms were accompanied by abnormal activations of these two brain areas. Therefore, the aim of the study was to identify a putative, resting-state intrinsically connected cerebello-amygdaloid network.

**Methods:**

ICA-based analysis was performed on brain resting-state functional images of 15 volunteers.

**Results:**

The first ICA spatial component corresponded to a circuit including: dentate nuclei, lobules VI and VIII, the basolateral amygdala, the substantia nigra, the posterior insula, claustrum and the parietal opercule.

**Conclusion:**

A new intrinsically connected network linking cerebellum and amygdala is described, which could be in charge of sensorimotor, emotional and motivational integration of somesthesic stimuli before recruiting more specialized circuits such as ventral striatum or attentional and salience networks.

## Background

Cerebellum partakes in several resting-state, intrinsically connected networks (ICNs) including: default-mode, right and left executive, limbic salience, dorsal attentional and motor networks (Habas et al. [1]; Sang et al. [2]; Brissanden et al. [3]). Cerebellum is assumed to act as a general modulator optimizing and automating affective, cognitive and motor processes, likely by generating internal models. Moreover, functional coherence was found between cerebellum (lobules I-V and hemispheric part of lobule VIII-IX) and amygdala (Sang et al. [2]). Histological tracing and evoked potential in monkey and in cat, demonstrated connections between fastigial nuclei and basolateral amygdaloid nuclei (Heath and Harper [4]; Snider and Maiti, [5]). However, no ICN encompasses amygdala.

Therefore, resting-state data of a previous study (Paris dataset; Habas et al. [1]) was reassessed investigating a potential ICN functionnally interconnecting the cerebellum and the amygdaloid nucleus. Our previous investigation only aimed at identifying cerebellar zones functionnaly linked to known ICNs, using binary mask of the five afore mentionned ICNs, and did not seek for new cerebello-cortical circuits. In the current study, we searched for a putative cerebello-amygdaloid network compatible with previous anatomical and fMRI data.

## Methods

### Subjects

Fifteen right-handed subjects (ages: 19–40; mean age: 26.5; nine females) were scanned, being requested to remain still, motionless and eyes closed. They gave their written informed consent, and had no history of cardiovascular nor neurologic disease.

### Acquisition sequences

Resting-state fMRI was performed on a whole-body 3 T scanner (Signa Horizon; General Electric Healthcare, Milwaukee, Wis.). An eight-channel head coil was utilized. Thirty-two contiguous axial T2*-weighted gradient echo-planar images (echo time 40 ms, repetition time 2500 ms, field of view 30 × 30, matrix 128 × 128 mm zero filled to 256 × 256 mm, slice thickness 4 mm, no interslice gap), were acquired to encompass the whole brain. Two hundred-sixteen volumes were acquired with four “dummy” volumes recorded at the start of the session to allow for steady-state magnetization.

### Post-processing

Group-level analysis (*N* = 15) was carried out, using probabilistic, temporal concatenation-based Independent Component Analysis (ICA) implemented in the MELODIC software, part of FSL (http://www.fmrib.ox.ac.uk/fsl). Images were first preprocessed (spatially and temporally smoothed, motion corrected, coregistered using a template brain of Montreal Neurological institute (MNI). Afterwards, ICA analysis with a usual posterior probability threshold equal to 0.05, was applied to the data. ICNs were identified by visual inspection. Clusters were localized using the MNI template and probabilistic atlas of the cerebellum provided by the FSLview interface (http://www.diedrichsenlab.org/imaging/propatlas.html).

## Results

### Functional data

**Fig. 1:**
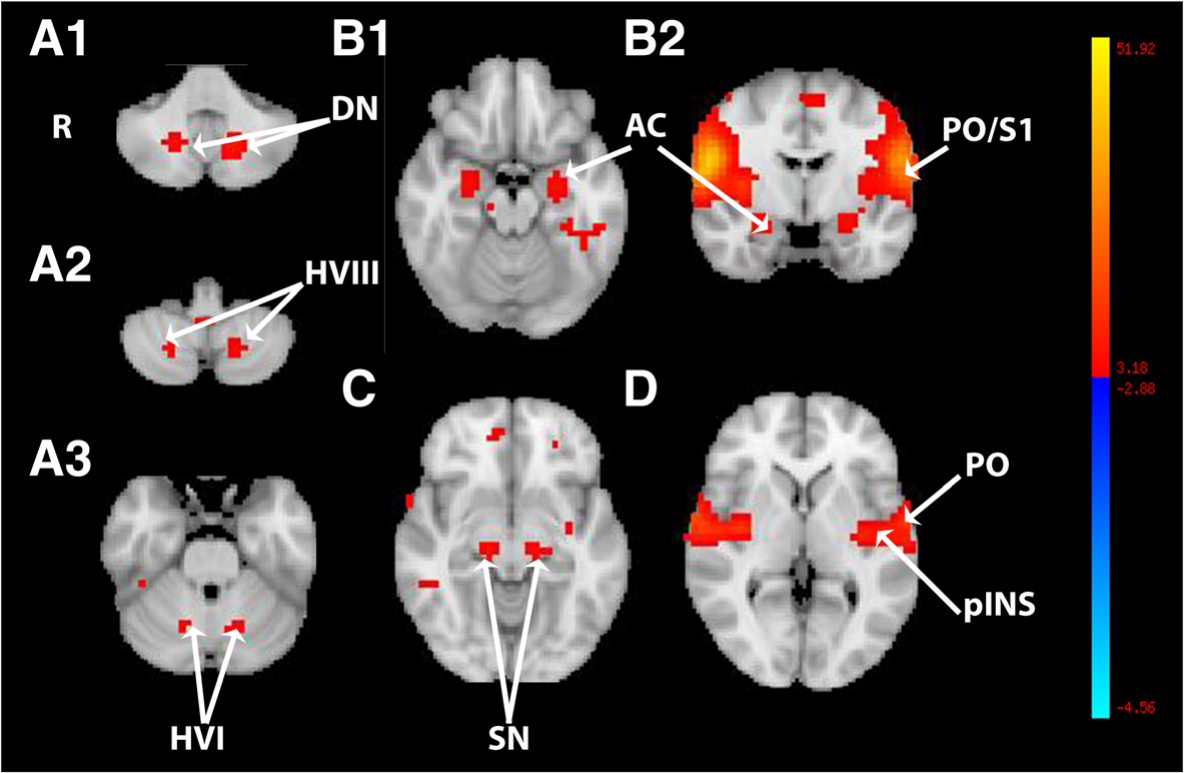
Multisubject (*N* = 15) thresholded spatial map (*p* > 0.5) of the first component computed by ICA analysis and showing the cerebello-amygdaloid intrinsically connected network. **A1-A3**. axial slices passing through the cerebellum (DN, dentate nuclei; HVI-HVIII, hemispheres of lobules VI and VIII). **B**. Axial (B1) and coronal (B2) slices passing through the encephalon and the amydaloid complex (AC). **C**. axial slice passing through the substantia nigra (SN). **D**. axial slice passing through the insula (INS) and parietal opercule (PO). The The bar from blue to yellow represents the z-value

The first generated ICA component corresponded to a bilateral (left hemispheric predominance) intrinsically connected network including (Fig. 1 and Table 1): cerebellum (dentate nuclei, hemispheres of lobule VIIIa and VI), substantia nigra with a possible extension to the subthalamic area, cerebral amydalae (mainly the laterobasal nucleus), the posterior insula with a likely extension to the claustrum, and the parietal opercule (SII) and SI. This circuit was clearly distinct from the other resting-state networks also identified previously in this study (Habas et al. [1]). A cluster was also noticed on the lobule IX. Further networks identified were found: default-mode, right and left executive, salience, visual, auditory and motor networks, identical with the ICNs reported in our previous study (Paris dataset; Habas et al. [1]).

**Table 1:** Cluster localization

		Right side	Left side
		(x,y,z)^a^	(x,y,z)^a^
Cerebellum	Dentate nucleus	17.20,-61.35,-35.00	−14.74,-64.65;-35.00
	Lobule VIIIa(/b)	21.86; −64.65;-49.00	−18.90;-65.75;-49.00
	Lobule VI	15.49;-63.78;-27.00	−16.45;-65.98;-27.00
Substantia nigra		13.30,-21.69;-9.00	−10.82,-20.58;-7.00
Cerebral amygdala	Latero-basal nucleus	25.77;-4.06;-21.00	−24.89;-7.36;-21.00
Posterior insula		38.87;-3,19;-5.00	−40,20;-9.80,-1.00s
Parietal opercule	SI/SII	60.78;-7.60;11.00	−60.40;-10.90;11.00

^a^MNI coordinates

## Discussion

We described a resting-state, bilateral limbic cerebello-amygdaloid network (CAN), using ICA analysis. First, this result is in agreement with anatomical data (reviewed in: Nieuwenhuys et al. [6]) in animals concerning amygdaloid interconnections with cerebellum, substantia nigra and insula, especially the posterior insula (Shi and Cassell [7]). Second, in animals, the fastigial nucleus projects to the amygdala (Heath and Harper [4]). In the current study, functional coherence was found between amygdala and dentate nuclei. In humans, the main output from the cerebellar cortex originates from dentate nuclei. However, a specific fastigial contribution cannot be ruled out, since the MRI spatial resolution can be too low to detect fastigial BOLD signal. Third, in humans, functional coherence was found between, cerebellum and amydala (Seng et al. [2]; Mischra et al. [8]; Roy et al. [9]), amygdala and insula (Roy et al. [9]; Robinson et al. [10]), insula and cerebellum, as well as parietal operculum (Cauda et al. [11]). However, CAN comprises the cerebellar lobule VI which belongs to motor, and, to a lesser extent, to salience and cognitive ICNs (Habas et al. [1]), and the posterior insula, and not the anterior insula which contributes to salience and right ventral attentional ICNs. The posterior insula interconnected with parietal opercule and sensorimotor cortex, is involved in the pain matrix (pain localization and valuation of pain intensity), and sensorimotor processing (Uddin [12]). We must emphasize that CAN is clearly distinct from salience network. Fourth, Morris et al. [13] found amygdaloid and cerebellar coactivation during presentation of fearful faces in a PETscan study. Moreover, some pathological neuropsychological states are accompanied by dysfunction of amygdaloid-based circuit including posterior insula and cerebellum (for instance, in case of generalized anxiety disorder: Roy et al. [14]. These authors ascribed the amygdala-cerebellar functional alteration to impairment in fear processing. Fifth, CAN also includes substantia nigra with a possible extention to the subthalamic area. Substantia nigra, and the amygdala, partake in the ventral striatum implicated in emotion, reward and motivational processing. The cluster within the substantia nigra extended within the subthalamic region so that it could be possible that subthalamic nucleus would be part of CAN. In this vein, the cerebellum participates in a cerebello-striato-subthalamo-ponto-cerebellar loop (Hoschi et al. [15]; Bostan et al. [16]; Pelzer et al. [17]). However, to our knowledge, no direct connections have been observed between the cerebellum and substantia nigra.

Based on these data, it can been hypothesized that the CAN, as an ICN, would be involved in bottom-up sensory processing (SI/SII and the posterior insula) further associated with: emotional evaluation (amygdala), reward and motivational appraisal (substantia nigra) and motor preparation (cerebellum). One example would be painful stimuli whose localization and intensity, on one hand, and emotional content, on the other hand, are evaluated in SII/insula, and amygdala, respectivelly, and which can generate aversive behavior (ventral striatum) and withdrawal movement (cerebellum). Moreover, all these neural nodes can also recruit other networks such as: salience and ventral attentional network via the posterior insular efferents to the anterior insula, the whole ventral striatum via the substantia nigra and amygdala, and the cerebellum as a polymodal hub for all ICNs.

Finally, a largest sampling and the utilization of other statistical methods, such as generalized canonical correlation analysis, could estimate, in further studies, the robustness of the present results.

## Conclusion

CAN could constitute an ICN devoted to rapid multimodal evaluation of somesthesic stimuli before selectively dispatching inputs to more specialized circuits.

## Abbreviations

CAN: cerebello-amygdaloid network
ICN: instrinsically connected network.s
